# Efficacy and tolerability of scalp cooling in preventing alopecia during (neo)adjuvant chemotherapy for breast cancer

**DOI:** 10.3389/pore.2026.1612403

**Published:** 2026-06-26

**Authors:** Dóra Sántha, Ágnes Dobi, Máté Iványi, Domonkos Válóczi, Zoltán Varga, Judit Oláh, Aliz Nikolényi

**Affiliations:** Department of Oncotherapy University of Szeged, Albert Szent-Györgyi Health Centre, Szeged, Hungary

**Keywords:** adverse effect, alopecia, breast cancer, chemotherapy, scalp cooling

## Abstract

**Background:**

Modern chemotherapies substantially improve survival rates in patients with breast cancer; however, its associated adverse effects can markedly diminish quality of life. Chemotherapy-induced alopecia is among the most common and distressing side effects. This study aimed to evaluate the efficacy and tolerability of the PAXMAN® scalp cooling system in preventing chemotherapy-induced alopecia among women with breast cancer receiving (neo)adjuvant chemotherapy.

**Methods:**

We conducted a prospective clinical study enrolling patients with early-stage breast cancer who received Orbis PAXMAN® scalp cooling during (neo)adjuvant chemotherapy. The extent of alopecia was assessed at the discontinuation of scalp cooling using the CTCAE v5 scale. Patient characteristics—age, menopausal status, hair length, color, quality, and prior treatments (e.g., dyeing, perming), as well as skin type, eye color, comorbidities, alcohol consumption, smoking, and chemotherapy regimen—were recorded. Associations between patient-related factors and the severity of alopecia were analyzed. Patients evaluated pain, cold sensation, and overall tolerability of the cooling process using a visual analogue scale.

**Results:**

Fifty female patients were enrolled. The mean age was 52 years (range 31–77); 27 patients were premenopausal and 23 were postmenopausal. Forty-two patients received an anthracycline-based regimen, while eight received anthracycline-free chemotherapy. Treatment discontinuation occurred in 31 cases due to severe alopecia and in one case due to intolerable pain. Grade 1 alopecia was observed in 18 patients (36%). The cooling cap combined with a 60-min post-cooling period demonstrated a promising success rate among patients receiving anthracycline-free regimens (69%), whereas efficacy was less favorable in anthracycline-based protocols (37.5%). Among the examined patient characteristics, only the presence of comorbidities showed a significant association with the severity of alopecia (p = 0.028). Most patients found the scalp cooling system comfortable; the mean scalp pain score was 2.17 and the mean cold sensation score was 3.9 on the visual analogue scale.

**Conclusion:**

The Orbis PAXMAN® scalp cooling system is an effective and well-tolerated method for preventing chemotherapy-induced alopecia, particularly in patients receiving anthracycline-free regimens. The presence of comorbidities significantly increased the risk of severe alopecia. While this study confirms that the efficacy of scalp cooling is strongly influenced by the chemotherapy regimen, further research is warranted to optimize patient selection and improve outcomes, especially for those undergoing anthracycline-based therapies.

## Introduction

According to the WHO Global Cancer Burden data, approximately 20 million new cases and 9.74 million deaths could be attributed to malignancies in 2022, while projections for 2045 estimate 32.6 million new cases and 16.9 million deaths. The incidence of breast cancer is expected to increase by 46% from 2.3 million in 2022 to 3.36 million in 2045 [1]. By 2040, the number of patients requiring first-line chemotherapy is predicted to have risen by 53%, with breast cancer representing the second most common indication [2].

Breast cancer and its therapeutic sequelae exert profound effects on personality, self-esteem, and self-perception in affected women [3]. Among the adverse consequences, chemotherapy-induced alopecia (CIA) represents one of the most common and distressing toxicities, with its prevalence approaching 70% [4]. Previous studies have demonstrated that the psychological burden of alopecia may, in some instances, exceed that of mastectomy [5–8]. Furthermore, women experiencing CIA frequently report altered social interactions, ranging from increased sympathy to stigmatization or rejection [9–11]. The risk of CIA is strongly influenced by the type, dose, and number of cytotoxic agents administered [12]. Antimicrotubule agents, such as paclitaxel, topoisomerase inhibitors including doxorubicin and epirubicin, and alkylating agents, such as cyclophosphamide are associated with the highest rates of alopecia (80%, 60%–100%, and 60%, respectively). By contrast, antimetabolites, such as 5-fluorouracil and leucovorin are associated with a moderate risk, ranging between 10% and 50% [13, 14].

Given that approximately 85%–90% of hair follicles are in the anagen phase, they are particularly vulnerable to cytotoxic damage [15]. Hair loss generally manifests within days to weeks after the initiation of chemotherapy and may persist for 1–3 months following treatment completion. Regrown hair frequently differs in color or texture compared to baseline [16], while in certain cases alopecia may be permanent or only partially reversible, even years after the therapy [17–19].

Scalp cooling (SC) or cryotherapy (using cold air, gel packs, or electronically cooled caps) has been widely implemented since the 1970s as an evidence-based strategy to prevent or mitigate CIA [20].

Scalp cooling exerts its protective effect through two principal mechanisms: (1) reduction of the local concentration of chemotherapeutic agents, and (2) suppression of hair follicle metabolism. Local temperature reduction induces vasoconstriction, thereby limiting drug delivery to the scalp, while concomitant decrease in follicular metabolic activity reduces the susceptibility of hair follicle cells to the antimitotic and antimetabolic effects of systemic therapy [10, 21]. Additional mechanisms have also been described, including induction of cell cycle arrest in the G0/G1 phase, increased accumulation of the stress-protective heat shock protein HSP70, and attenuation of apoptosis, all of which contribute to follicular preservation during chemotherapy [22]. A systematic review [10] and a meta-analysis of randomized controlled trials [23] have conclusively demonstrated the significant efficacy of scalp cooling in reducing chemotherapy-induced alopecia among patients with solid tumors.

While the highest efficacy in hair preservation is observed with taxane-containing regimens, scalp cooling confers a clinically relevant benefit even in the context of anthracycline therapy, despite lower overall effectiveness. According to the literature, scalp cooling could prevent hair loss in 65%–100% of patients treated with a taxane-based, anthracycline-free therapy [24–27], while a substantially lower, 36%–60% success rate was demonstrated with anthracycline-containing regimens [23, 28, 29].

Regarding adverse effects, some studies [3] reported rates as high as 86.8%, ranging from commonly occurring headache or cold sensation to less frequently observed nausea, paresthesia, and skin ulcers.

This prospective study was designed to explore the effectiveness of a scalp cooling system with a 60-min post-infusion cooling period in early-stage breast cancer patients receiving adjuvant or neoadjuvant chemotherapy, and to identify patient- or disease-related factors predictive of successful hair preservation, thereby improving criteria for optimal patient selection. An additional objective of the study was to assess adverse effects associated with scalp cooling.

## Materials and methods

Prospective data collection was carried out to evaluate the effectiveness and tolerability of the Orbis PAXMAN® scalp cooling device in women with early breast cancer undergoing (neo)adjuvant chemotherapy at the Department of Oncotherapy, University of Szeged. The study was approved by the local Ethics Committee (61/2023-SZTE RKEB). The present report summarizes findings from the first 50 patients enrolled.

The application of the scalp cooling device was offered to all eligible women who met the inclusion and exclusion criteria and for whom the multidisciplinary breast tumor board recommended (neo)adjuvant chemotherapy. Prior to enrollment, each patient received detailed study information and provided written informed consent. Within this prospective cohort, analyses were performed on women aged 18–80 years with stage I–III early breast cancer and an ECOG performance status of 0–1, who underwent (neo)adjuvant chemotherapy regimens summarized in Table 1.

**TABLE 1:** Types, doses, and regimens of neoadjuvant and adjuvant systemic therapies.

Type of chemotherapy	Dosage	Treatment schedule
Doxorubicin + cyclophosphamide	60 mg/m^2^ + 600 mg/m^2^	4 cycles every 3 weeks
Epirubicin + cyclophosphamide	90 mg/m^2^ + 600 mg/m^2^	4 cycles every 3 weeks
Docetaxel + cyclophosphamide	75 mg/m^2^ + 600 mg/m^2^	6 cycles every 3 weeks
Paclitaxel +/- trastuzumab	80 mg/m^2^	12 cycles weekly
Paclitaxel	175 mg/m^2^	4 cycles every 3 weeks
docetaxel+/- trastuzumab +/- pertuzumab	75 mg/m^2^	4 cycles every 3 weeks
Paclitaxel + carboplatin	80 mg/m^2^+ AUC 1.5	12 cycles weekly
Paclitaxel + carboplatin	175 mg/m^2^+ AUC 6	4 cycles every 3 weeks
Cyclophosphamide + methotrexate + fluorouracil	100 mg/m^2^ + 40 mg/m^2^ + 600 mg/m^2^	6 cycles on day 1 and 8 of 28-day cycles

Abbreviations: AUC, Area Under the Curve; mg, milligram.

Exclusion criteria included a history of previous chemotherapy and autoimmune disorders affecting the hair, such as alopecia areata, systemic lupus erythematosus associated with alopecia, or androgenic alopecia. Patients with prior whole-brain irradiation or clinically significant liver disease (e.g., active viral hepatitis) were not eligible. Abnormal liver function tests exceeding 1.5 times the upper limit of normal, including alkaline phosphatase, aspartate aminotransferase, and total bilirubin, also precluded participation. Individuals with Gilbert’s disease, however, were permitted to enroll. Additional exclusion criteria comprised clinically significant renal impairment, untreated or poorly controlled thyroid dysfunction, severe concurrent infections, or other serious comorbidities that could interfere with the planned systemic therapy. Patients with a history of migraine, cold agglutinin disease, cryoglobulinemia, or silicone allergy were likewise considered ineligible.

Before enrollment, patients received a Patient Information Sheet regarding the use of the scalp cooling device, as well as guidance on hair care during the treatment and between chemotherapy cycles. These recommendations were consistent with the manufacturer’s guidelines.

A dermatological examination was performed prior to the first treatment. The assessment included hair type (straight-type1, wavy-type2, curly-type3, kinky-type4), quality (fine or thick), length (<5 cm or ≥5 cm), color (blonde, brown, black, grey, or red), prior chemical hair treatments (coloring, dyeing, perming, straightening), eye color (blue, green, brown, black), Fitzpatrick skin type (I–VI), degree of UV-induced skin damage (none, minimal, moderate, significant). Baseline documentation also covered patient characteristics (age, menopausal status, body weight, height, body mass index), disease-related factors (tumor subtype by immunohistochemistry, tumor stage), smoking, alcohol consumption, and comorbidities.

Smoking status was categorized as never, former, or current smoker. Current smokers were defined as patients who had smoked at least 100 cigarettes during their lifetime and were actively smoking at the time of treatment initiation; former smokers had smoked at least 100 cigarettes during their lifetime but were not smoking at treatment initiation. In the analysis, ever smokers (current and former) were compared with never smokers. Alcohol consumption was categorized as none and current use. Current alcohol use was further stratified into low and high consumption levels based on weekly alcohol intake. Low consumption was defined as ≤10 units per week, while high consumption was defined as >10 units per week. Alcohol intake was estimated based on patient-reported average weekly consumption (1 unit = 10 g ethanol).

Comorbidities were defined as pre-existing, physician-diagnosed chronic medical conditions under active medical treatment at the time of oncological diagnosis. Comorbidity categories were defined based on prior literature suggesting an increased risk of androgenetic alopecia in patients with metabolic syndrome–related conditions, including hypertension, diabetes mellitus, and dyslipidemia. [30]. Accordingly, three categories were established: no comorbidity, metabolic syndrome–related comorbidities (hypertension, diabetes mellitus, and/or dyslipidemia), and other comorbidities. In patients with multiple conditions, those presenting with any metabolic syndrome–related comorbidity were classified into this category for statistical analysis.

Standardized photographs of the hair were taken from three angles before the first cycle of chemotherapy, 3 weeks after the first chemotherapy, and 3 weeks after the completion of treatment.

Before chemotherapy, the Orbis PAXMAN® scalp cooling device was pre-cooled to operating temperature, with the cooling fluid maintained at −4 °C. Meanwhile, patients prepared their hair by smoothing it to reduce volume and remove air pockets, moistening it to improve conductivity, and applying conditioner to facilitate cap removal after post-cooling. Thirty minutes prior to chemotherapy, a suitably sized cap was fitted. Circulating coolant reduced scalp temperature to 18 °C–22 °C (64–72°F) and maintained it throughout the infusion, thereby inducing vasoconstriction and limiting cytotoxic drug delivery to hair follicles. To further mitigate peak exposure of chemotherapeutic agents, cooling was maintained for 60 min following chemotherapy (post-infusion cooling time, POIC).

The primary aim of the study was to assess the efficacy and tolerability of scalp cooling. The primary endpoint was the degree of alopecia, assessed according to CTCAE v5. Grade 1 alopecia was defined as <50% hair loss, not clearly visible from a distance and concealable with alternative hairstyles, without requiring wigs or hair prostheses. Grade 2 alopecia was defined as ≥50% hair loss, readily noticeable to others, requiring a wig or hair replacement for concealment, and associated with psychosocial impact.

Correlations were examined between the degree of hair loss and both the type of chemotherapy administered and patient-related characteristics. The primary endpoint was evaluated at the time the patient discontinued scalp cooling, either upon completion of (neo)adjuvant therapy or earlier if the patient chose to suspend the procedure.

When comparing alopecia grade and individual patient characteristics, an independent samples t-test was used for continuous variables, while for categorical variables a chi-square test was applied. Statistical data analysis was performed using SPSS version 26.0.

Secondary endpoints focused on tolerability of the cooling. After chemotherapy sessions, patients rated scalp pain and cold sensation experienced during cooling on a visual analogue scale (VAS) with 1–10 scoring options. The Massey adapted scoring system was applied to summarize device-related comfort as follows: 1. very comfortable, 2. reasonably comfortable, 3. comfortable, 4. uncomfortable, and 5. very uncomfortable [15]. Data from the entire study population were analyzed irrespective of chemotherapy regimen, as previous studies suggest that adverse events associated with scalp cooling are primarily related to the cooling procedure itself and do not appear to significantly differ across chemotherapy types [31].

## Results

### Patient and disease characteristics

Between 29 June 2023, and 30 March 2025, a total of 50 patients with early breast cancer were enrolled in our study. Patient, disease, and treatment characteristics are summarized in Table 2. At diagnosis, the mean age was 52 years (range, 31.7–77.2); 27 patients (54%) were premenopausal and 23 (46%) were postmenopausal. The mean body mass index (BMI) was 26.08 kg/m^2^ (range, 18.0–37.18); 22 patients had a BMI below 25 kg/m^2^, while the majority were overweight.

**TABLE 2:** Baseline patient, disease, and treatment characteristics.

Characteristics		Patients no. (%)
Mean age (years) (range)	​	52.01 (31.7–77.2)
Menopausal status	Premenopausal	27 (54.0)
	Postmenopausal	23 (46.0)
IHC subtype of breast cancer	ER and/or PR+, HER2-	20 (40.0)
	ER and/or PR+, HER2+	11 (22.0)
	ER and PR-, HER2+	2 (4.0)
	ER and PR-, HER2-	17 (34.0)
Type of chemotherapy	Anthracycline free regimen	8 (16.0)
	Anthracycline containing regimen	42 (84.0)
Chemotherapy regimen	AC-TAX (±HER2 targeted therapy)	27 (54.0)
	AC-TAX + CBP	12 (24.0)
	TC	4 (8.0)
	TAX (±HER2 targeted therapy)	3 (6.0)
	CMF	1 (2.0)
	AC-T (±HER2 targeted therapy)	3 (6.0)
BMI (kg/m^2^)	<25	22 (44.0)
	≥25	28 (56.0)
Hair type	Type 1 or 2	46 (92.0)
	Type 3 or 4	4 (8.0)
Hair thickness	Thin hair	16 (32.0)
	Thick hair	34 (68.0)
Hair color	Blonde	8 (16.0)
	Brown	33 (66.0)
	Black	1 (2.0)
	Grey	8 (16.0)
Hair length	Short (<5 cm)	15 (30.0)
	Long (≥5 cm)	35 (70.0)
Eye color	Blue	22 (44.0)
	Green	5 (10.0)
	brown	23 (36.0)
Hair processing (curling, dyeing, straightening)	Yes	34 (68.0)
	No	16 (32.0)
Fitzpatrick skin type	1	1 (2.0)
	2	14 (28.0)
	3	34 (68.0)
	4	1 (2.0)
Seborrheic keratosis	No	12 (24.0)
	Yes	38 (76.0)
Sun damage of skin	None	2 (4.0)
	Minimal	16 (32.0)
	Moderate	21 (42.0)
	Severe	11 (22.0)
Comorbidities	None	23 (46.0)
	Hypertension/diabetes mellitus/dyslipidaemia	19 (38.0)
	Other	8 (16.0)
Alcohol consumption	No	48 (96.0)
	Low consumption	2 (4.0)
	High consumption	0 (0.0)
Smoking	Never	37 (74.0)
	Ever	13 (26.0)

Abbreviations: A, doxorubicin; BMI, body mass index; C, cyclophosphamide; CBP, carboplatin; ER, estrogen receptor; F, 5-fluorouracil; HER2, Human Epidermal Growth Factor Receptor 2; IHC, immunohistochemistry; M, methotrexate; PR, progesterone receptor; TAX, paclitaxel; T, docetaxel.

At enrollment, the distribution of patients by disease stage according to the American Joint Committee on Cancer (AJCC) tumor-node-metastasis (TNM) staging system was as follows: 7 patients with stage I breast cancer, 33 with stage II disease, and 8 with stage III breast cancer; all received neoadjuvant or adjuvant systemic therapy. One patient was not categorized by TNM staging system due to prior axillary block dissection performed during the treatment of a previous breast cancer (cNx), and another one due to occult breast cancer (cTx). Most patients (74%) had HER2-negative disease, and 17 patients were treated for triple-negative breast cancer.

Regarding chemotherapy, the most common regimen was a combination of anthracycline followed by paclitaxel (54%), with the addition of carboplatin in a further 12 cases (24%). Four patients were treated with a combination of docetaxel–cyclophosphamide, 3 patients received anthracycline followed by docetaxel, 3 patients were given weekly paclitaxel, and 1 patient received a triplet combination of cyclophosphamide–methotrexate–5-fluorouracil (CMF). Overall, 8 patients received anthracycline-free regimens, whereas 42 patients were treated with anthracycline-containing regimens. All 13 patients with HER2-positive breast cancer received HER2-targeted therapy in addition to chemotherapy.

Most patients had straight or wavy hair (type 1 or 2) (46/50), and in 34 cases the hair was classified as thick by the dermatologist. The majority (35/50) had long hair. Hair color was brown in 33 patients, blonde in 8, grey in 8, and black in 1. Eye color distribution was as follows: brown in 23 patients, blue in 22, and green in 5. A substantial proportion (34/50) reported previous hair treatments with potential negative effects on hair quality, such as ammonium-containing dyes, regular straightening, or curling.

Based on the dermatological examination before the treatment, most patients had Fitzpatrick skin type II or III—that is, fair skin with blond or brown hair—characterized by frequent or occasional sunburn upon sun exposure. Sun-induced skin damage was predominantly minimal (16/50) or moderate (21/50).

Alcohol consumption was uncommon among the patients enrolled (2/50, 4.0%), and both patients were classified as low consumers. Of the 50 individuals, 37 (74.0%) were never smokers, while 13 (26.0%) were current or former smokers. Comorbidities were present in 27/50 patients (54.0%), all of whom were receiving active pharmacological treatment for their underlying conditions. Among these, 19/50 patients (38.0%) had metabolic syndrome–related comorbidities; all of these patients had hypertension, with one patient also having diabetes mellitus and one patient dyslipidemia. The remaining 8/50 patients (16.0%) were classified into the other comorbidity group, which included hypothyroidism (n = 5), asthma (n = 1), depression (n = 1), and gastroesophageal reflux disease (n = 1).

### Scalp cooling and hair loss

Thirty-one patients (62%) discontinued scalp cooling before completing the planned (neo)adjuvant therapy, most of them after 1 or 2 chemotherapy cycles, due to grade 2 hair loss; one additional patient withdrew because of intolerable scalp pain.

**FIGURE 1:**
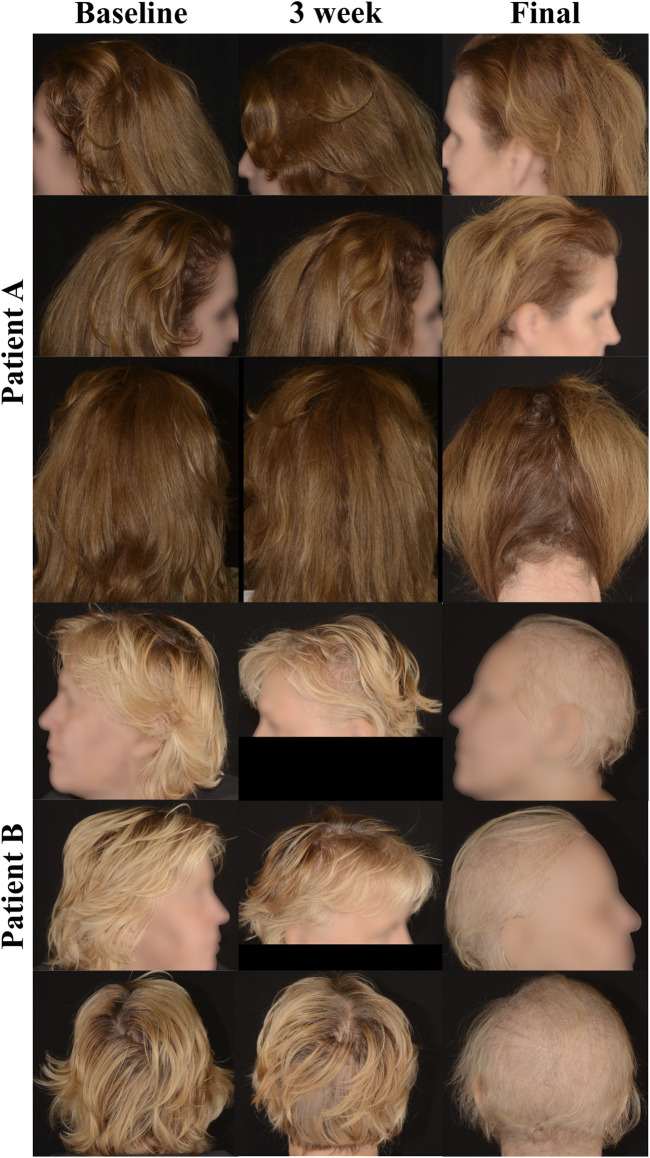
Photographs illustrating Grade 1 alopecia **(A)** and Grade 2 alopecia **(B)**.

Overall, joint evaluation conducted by physician and patient indicated that scalp cooling was successful in preventing chemotherapy-induced hair loss in 36% of patients; 18 of the 50 patients experienced <50% hair loss by the end of chemotherapy. Photographs of a patient with Grade 1 alopecia appear in Figure 1A and those of a patient with Grade 2 alopecia are shown in Figure 1B.

Associations between alopecia grade and patient characteristics are shown in Table 3.

**TABLE 3:** Relationship between hair loss and patient characteristics.

Patient characteristics		Patients no. with Gr.1 hair loss (%)	Patients no. with Gr.2 hair loss (%)	p-value
Mean age (years) ± SD	​	49.6 ± 11.1	53.4 ± 10.8	0.251
Menopausal status	Premenopausal	12 (44.4)	15 (55.6)	0.146
	Postmenopausal	6 (26.1)	17 (73.9)	
BMI (kg/m^2^)	<25	10 (45.5)	12 (54.5)	0.249
	≥25	8 (28.6)	20 (71.4)	
Hair type	Type 1 or 2	15 (32.6)	31 (67.4)	0.127
	Type 3 or 4	3 (75.0)	1 (25.0)	
Hair thickness	Thin hair	4 (25.0)	12 (75)	0.215
	Thick hair	14 (41.2)	20 (58.8)	
Hair color	Blonde	2 (25)	6 (75)	0.426
	Brown	13 (39.4)	20 (60.6)	
	Black	1 (100)	0 (0)	
	Grey	2 (25)	6 (75)	
Hair length	Short (<5 cm)	3 (20)	12 (80)	0.109
	Long (≥5 cm)	15 (42.9)	20 (57.1)	
Eye color	Blue	7 (31.8)	15 (68.2)	0.861
	Green	2 (40.0)	3 (60.0)	
	Brown	9 (39.1)	14 (60.9)	
Hair processing (curling, dyeing, straightening)	Yes	10 (29.4)	24 (70.6)	0.136
	No	8 (50)	8 (50)	
Fitzpatrick skin type	1	1 (100.0)	0 (0.0)	0.420
	2	6 (42.9)	8 (57.1)	
	3	11 (32.4)	23 (67.6)	
	4	0 (0.0)	1 (100.0)	
Seborrheic keratosis	No	15 (39.5)	23 (60.5)	0.291
	Yes	3 (25)	9 (75)	
Sun damage of skin	None	1 (50)	1 (50)	0.969
	Minimal	6 (37.5)	10 (62.5)	
	Moderate	7 (33.3)	14 (66.7)	
	Severe	4 (36.4)	7 (63.6)	
Comorbidities	None	12 (52.2)	11 (47.8)	0.050
	Hypertension/diabetes mellitus/dyslipidaemia	3 (15.8)	16 (84.2)	
	Other	3 (37.5)	5 (62.5%)	
Alcohol consumption	No	16 (33.3)	32 66.7)	0.125
	Low consumption	2 (100)	0 (0)	
	High consumption	0 (0)	0 (0)	
Smoking	Never	13 (35.1)	24 (64.9)	0.542
	Ever	5 (38.5)	8 (61.5)	

Abbreviations: BMI, body mass index.

The primary endpoint analysis revealed no statistically significant correlation between hair loss and patient-related factors, including age, menopausal status, BMI, hair quality, hair length, hair thickness, eye color, and history of hair manipulation. Fitzpatrick skin type and degree of UV-related skin damage showed no impact on the outcome of scalp cooling either. However, 21 (77.8%) out of 27 patients with systemic comorbidities developed Grade 2 alopecia compared to 11 (47.8%) out of 23 without systemic comorbidities. When stratified into three groups (no comorbidity, metabolic syndrome–related comorbidities, and other comorbidities), the proportion of patients with Grade 2 alopecia appeared highest in the metabolic syndrome–related group (16/19, 84.2%), compared to 5/8 (62.5%) in the other comorbidity group and 11/23 (47.8%) in patients without comorbidities (p = 0.05).

Table 4 illustrates the correlation between hair loss and the chemotherapy regimen administered. Among patients treated with anthracycline followed by paclitaxel, 66.7% experienced >50% hair loss, while among those receiving anthracycline followed by paclitaxel plus carboplatin, 75% developed Grade 2 alopecia. Grade 2 hair loss was also observed in three of four patients receiving docetaxel–cyclophosphamide combination therapy and in two of three patients on anthracycline–docetaxel therapy. In contrast, scalp cooling was effective in one patient treated with CMF and in three patients receiving weekly paclitaxel.

**TABLE 4:** Patient-reported hair loss according to chemotherapy type at scalp cooling discontinuation.

Chemotherapy type	Total (n)	Gr.1 hair loss n (%)	Gr.2 hair loss, n (%)
AC-TAX	27	9 (33.3)	18 (66.7)
AC-TAX/CBP	12	3 (25)	9 (75)
TC	4	1 (25)	3 (75)
TAX	3	3 (100)	0
CMF	1	1 (100)	0
AC-T	3	1 (33.3)	2 (66.5)

Abbreviations: A, doxorubicin; C, cyclophosphamide; CBP, carboplatin; M, methotrexate; F, 5-fluorouracil; TAX, paclitaxel; T, docetaxel.

As regards anthracycline-free regimens, favorable outcomes were observed in 5 of 8 patients (62.5%), with hair loss ≤50%, and no need for wigs. In contrast, the success rate was only 31% with anthracycline-containing regimens. This difference, however, did not reach statistical significance (p = 0.098).

### Adverse events

**FIGURE 2:**
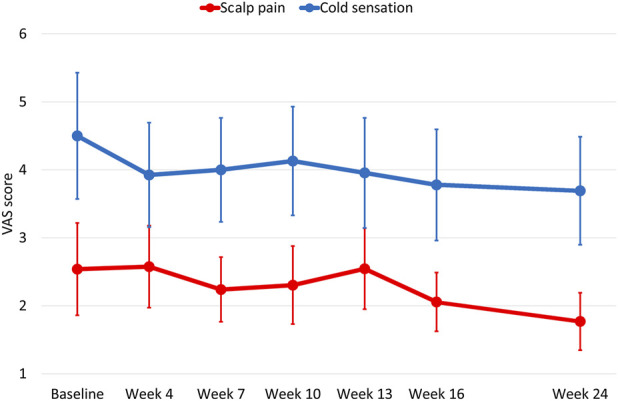
Visual-analog scale of head/scalp pain and feeling cold/shivering during scalp cooling.

During scalp cooling, 15 patients (30%) reported no pain at any time, while 16 patients reported a score of ≥5 on the visual analogue scale. The mean ± SD VAS score for pain showed a declining trend over the course of treatment, decreasing from 2.54 ± 2.37 at baseline to 2.06 ± 1.514 at week 16 and 1.77 ± 1.481 at week 24. A similar trend was observed for cold sensation: the mean ± SD VAS score decreased from 4.50 ± 3.25 at baseline to 3.78 ± 2.86 with minimal fluctuation, and further to 3.69 ± 2.78 by the end of the observation period (Figure 2). In contrast to scalp pain, cold sensation was reported by nearly all patients (except three), and about half reported a score of ≥5.

According to the adapted Massey scale, most patients rated scalp cooling as at least comfortable, with the mean score remaining stable throughout the treatment period. Nevertheless, 10 patients (20%) reported the cooling cap as uncomfortable on at least one occasion, and 3 patients (6%) described it as very uncomfortable.

## Discussion

This prospective study found that scalp cooling with a 60-min post-infusion application showed promising efficacy with anthracycline-free regimens, whereas it was less effective in preventing alopecia in patients with early breast cancer receiving anthracycline-based chemotherapy in the neoadjuvant or adjuvant setting.

In this cohort, the overall success rate of scalp cooling was 36%, but outcomes were less favorable with anthracycline-containing treatments: among patients receiving sequential AC followed by paclitaxel or docetaxel, only one-third maintained hair loss ≤50%, while in those treated with AC followed by paclitaxel plus carboplatin, the rate decreased to one-quarter. By contrast, anthracycline-free regimens combined with scalp cooling yielded more encouraging results, with only about one-third of patients experiencing significant alopecia; however, given the small sample size (n = 8), these findings should be interpreted with caution and may not be sufficient to support definitive conclusions.

Rugo et al., in a meta-analysis of 10 randomized trials (66% anthracycline-based regimens), demonstrated that scalp cooling reduces the risk of CIA by 46% [25]. Nevertheless, several studies reported less favorable outcomes with anthracycline-based therapies. Smetanay et al. observed a success rate of 36.4% [23], while Munzone et al. reported 46% [29], both applying longer post-infusion cooling of 90–120 min. In the SCALP randomized clinical trial, Nangia et al. found that ≤50% hair loss was achieved in 50.5% of patients overall, but in only 22% of those receiving anthracycline-containing regimens—consistent with the present findings [26].

Giarratano et al. highlighted the potential role of treatment sequencing: alopecia was markedly greater when anthracyclines preceded taxane compared with the reverse order (69% vs. 40%; p = 0.017) [24]. The possible biological explanation is that in taxane-first regimens, weekly administration and repeated cooling induce prolonged hypothermia, thereby reducing follicular metabolism and mitigating the subsequent cytotoxic effect of anthracyclines. In our cohort, all patients receiving anthracyclines subsequently underwent taxane ± carboplatin therapy. Consistent with previous reports, initiation of therapy with an anthracycline-based regimen in sequential anthracycline–taxane schedules was associated with poorer hair preservation. Prospective clinical trials have reliably demonstrated that scalp cooling prevents alopecia in 65%–100% of patients treated with taxane-based, anthracycline-free regimens [24–27]. The present analysis aligned with these data, as 5 of 8 patients maintained ≤50% hair loss. However, due to the small sample size of women treated without anthracyclines, no statistically significant association could be established. Still, the trend was evident: Grade 2 CIA occurred more frequently with anthracycline-based regimens (69% vs. 37.5%, p = 0.098).

Evidence from the literature also indicates that the duration of pre- and post-infusion cooling substantially influences efficacy. Pre-infusion cooling is most often applied for 5–30 min; however, some studies suggest extending it to 45 min to achieve optimal scalp temperature [32]. The optimal duration of post-infusion cooling remains uncertain, with recommendations in the literature ranging from 15 min to several hours. Overall, longer cooling periods tend to yield better outcomes for drugs with a long half-life [32], whereas for docetaxel, shorter cooling of only 45 min has been shown sufficient [33]. Data for epirubicin and cyclophosphamide remain inconsistent. In a randomized clinical trial, extending POIC to 150 min did not significantly reduce the need for head covering compared with 90 min; however, moderate to complete alopecia occurred less frequently with the longer cooling duration [32, 34]. In contrast, M. Mangesh et al. reported that the optimal cut-off for post-infusion cooling time is 150 min, as POIC>150 min after anthracycline-based chemotherapy was associated with a higher risk of alopecia compared with a shorter duration [20]. Carton et al., in a randomized pilot study, compared (i) cooling during treatment plus 30 min post-infusion and (ii) 30 min pre-infusion, cooling during treatment, and 2 h post-infusion cooling. They found no significant improvement with prolonged POIC, but it was associated with increased patient discomfort [33]. However, it should be noted that with epirubicin plus cyclophosphamide therapy, the proportion of patients with grade 0–1 alopecia was slightly higher with longer post-infusion cooling (40% vs. 47%, p = 0.41), whereas in paclitaxel-treated patients, better outcomes were observed with shorter post-infusion cooling (50% vs. 40%, p = 0.67). Because of heterogeneous clinical data, pre- and post-infusion cooling durations are often determined by local experience and logistical feasibilities, while also considering drug type, dose, and schedule. In our study, a protocol of 30 min pre-infusion and 60 min post-infusion cooling was chosen for all chemotherapy regimens, considering local resources. By contrast, most trials reporting more favorable results with anthracycline-based regimens used longer POIC of 90–120 min [20, 23, 26, 29, 32, 34, 35].

One of the primary objectives of our investigation was to identify correlations between CIA and predefined patient characteristics to improve patient selection, given the limited availability of scalp cooling systems. It was hypothesized that poorer hair quality—potentially influenced by age, skin type, hair texture, or previous chemical hair treatments—would reduce cooling efficacy. Prior studies have shown that age is the most consistent predictor, with lower efficacy observed in older or postmenopausal women [32, 36]. In the present analysis, most patient features were not predictive of Orbis Paxman system effectiveness, consistent with the findings of Pedersini et al. and Rugo et al [25, 37]. The incidence of alopecia did not differ by age, BMI, or menopausal status, nor by hair/skin characteristics, prior hair treatments, smoking, or alcohol consumption. However, comorbidities and concomitant medications may contribute to hair follicle damage, thereby promoting androgenetic alopecia or enhancing the negative effects of chemotherapy [38]. In this context, the association between metabolic and cardiovascular disorders and androgenetic alopecia has been increasingly supported by the literature, often preceding the clinically visible signs of alopecia. [30]. Several studies and meta-analyses have demonstrated a higher prevalence of hypertension among patients with alopecia, as well as other metabolic abnormalities, including dyslipidemia and insulin resistance. The underlying mechanisms are not yet fully clarified; however, a multifactorial pathogenesis is strongly suggested. These mechanisms include hormonal effects, as androgens—particularly dihydrotestosterone (DHT)—play a key role in the miniaturization of androgen-sensitive hair follicles [39]. In addition, through their effects on vascular smooth muscle cells, they may induce vascular alterations, leading to microcirculatory impairment and endothelial dysfunction. These changes may compromise blood flow to the hair follicles, resulting in reduced oxygen and nutrient supply. Furthermore, inflammation-related mechanisms associated with metabolic syndrome may also contribute to the development of alopecia. In this context, perifollicular microinflammation, along with inflammatory mediators—such as prostaglandin D2 and pro-inflammatory cytokines—has been implicated in hair follicle dysfunction [40]. In addition to hypertension, insulin resistance and hyperinsulinemia may further enhance local androgen production and increase the conversion of testosterone to DHT, thereby exacerbating hair loss [30]. In our study, the presence of comorbidities was also associated with reduced efficacy of scalp cooling. Notably, among the 50 patients, 19 had hypertension, of whom 16 experienced grade 2 alopecia, while only 3 patients developed grade 1 hair loss. These findings are in line with previous observations suggesting that hypertension-related pathophysiological alterations—such as microvascular impairment and endothelial dysfunction—may adversely affect hair follicle integrity and reduce the likelihood of successful hair preservation during chemotherapy.

Clinical trials have reported variable results regarding the tolerability of scalp cooling; although discontinuation due to adverse events is generally low, typically 10% or less [20, 24, 25, 37]. Consistent with these findings, the Orbis Paxman scalp cooling system was well tolerated in the present study. Regarding side effects, seventy percent of patients reported some pain during treatment, usually mild to moderate. Cold sensation occurred in all patients, with about half describing it as intense. Importantly, only one individual discontinued scalp cooling due to severe headache reported as an adverse event. Overall, 74% of participants described the device as comfortable, quite comfortable, or very comfortable, and no patient withdrew consent because of discomfort. These results are in line with the literature, showing good tolerability, acceptable comfort and adverse effects, and particularly low withdrawal rate [20, 24, 25, 37].

## Conclusion

In patients receiving anthracycline-free regimens for early breast cancer in the neoadjuvant or adjuvant setting, encouraging efficacy can be achieved with a 60-min post-infusion cooling period, whereas, when compared with a longer cooling duration, this shorter cooling time demonstrates lower efficacy in anthracycline-containing chemotherapies. No patient-related characteristics predictive of scalp cooling efficacy were identified in our study, except for comorbidities that may adversely affect hair follicle health. In particular, hypertension appeared to be associated with reduced efficacy. Therefore, adherence to hair care recommendations remains particularly important both during and between treatments.

Further investigations are warranted to optimize scalp cooling, including randomized evaluations of 90- and 120-min post-infusion cooling with anthracycline-based chemotherapy to assess the risk of both early and persistent alopecia.

### Limitations

This study has several limitations. First, the sample size was relatively small (n = 50), particularly in the subgroup receiving anthracycline-free regimens (n = 8), where heterogeneous treatments further limit the strength of conclusions. Second, the absence of a control group precludes direct estimation of the magnitude of benefit attributable to scalp cooling, and interpretation relies on indirect comparisons with published data. Third, a uniform post-infusion cooling time of 60 min was applied. As the optimal duration remains uncertain and varies across the literature, and longer cooling periods (90–120 min) are often used in anthracycline-based regimens, the shorter duration in this study may have contributed to the lower efficacy observed. Nevertheless, the observed hair preservation rate (36%) suggests that a proportion of patients may still derive clinically relevant benefit.

Taken together, these limitations highlight the need for larger, controlled studies with more homogeneous treatment groups and optimized cooling protocols to better define the role of scalp cooling across different chemotherapy regimens.

## Data Availability

The raw data supporting the conclusions of this article will be made available by the authors, without undue reservation.
